# Health Centre Surveys as a Potential Tool for Monitoring Malaria Epidemiology by Area and over Time

**DOI:** 10.1371/journal.pone.0026305

**Published:** 2011-11-04

**Authors:** Abraham R. Oduro, Kalifa A. Bojang, David J. Conway, Tumani Corrah, Brian M. Greenwood, David Schellenberg

**Affiliations:** 1 Medical Research Council Laboratories, Banjul, The Gambia; 2 London School of Hygiene and Tropical Medicine, London, United Kingdom; University of Western Australia, Australia

## Abstract

**Background:**

Presently, many malaria control programmes use health facility data to evaluate the impact of their interventions. Facility-based malaria data, although useful, have problems with completeness, validity and representativeness and reliance on routinely collected health facility data might undermine demonstration of the magnitude of the impact of the recent scaleups of malaria interventions. To determine whether carefully conducted health centre surveys can be reliable means of monitoring area specific malaria epidemiology, we have compared malaria specific indices obtained from surveys in health centres with indices obtained from cross-sectional surveys conducted in their catchment communities.

**Methods:**

A series of age stratified, seasonal, cross-sectional surveys were conducted during the peak malaria transmission season in 2008 and during the following dry season in 2009 in six ecologically diverse areas in The Gambia. Participants were patients who attended the health centres plus a representative sample from the catchment villages of these health facilities. Parasitaemia, anaemia, attributable proportion of fever and anti-MSP1-_19_ antibody seroprevalence were compared in the health facility attendees and community participants.

**Results:**

A total of 16,230 subjects completed the study; approximately half participated in the health centre surveys and half in the wet season surveys. Data from both the health centre and community surveys showed that malaria endemicity in The Gambia is now low, heterogeneous and seasonal. In the wet season, parasitaemia, seroprevalence and fever prevalence were higher in subjects seen in the health centres than in the community surveys. Age patterns of parasitaemia, attributable proportions of fever and seroprevalence rates were similar in subjects who participated in the community and health centre surveys.

**Conclusion:**

Health centre surveys have potential as a surveillance tool for evaluating area specific malaria control activities and for monitoring changes in local malaria epidemiology over time.

## Introduction

Success in malaria control is leading to significant changes in the epidemiology of malaria infection and disease in some endemic areas [1]–[3]. It is important that these changes are monitored adequately in order to track the progress that is being made in control over time. There is also a need for current data on populations at risk and the proportion of malaria cases in relation to other illnesses [4]–[5]. This information is necessary for the design and implementation of new interventions for malaria and other febrile illnesses and to target interventions where they are needed most. Realistic estimates of the current malaria burden are required to establish impact on health and development and to estimate coverage with the preventive tools that are being deployed [6].

Presently, many control programmes use health facility data to evaluate the impact of their malaria interventions [1]. Facility-based data have problems with completeness, validity and representativeness because of the mode of collection, analysis and reporting of facility data in many high burden malaria countries [7]–[9]. This situation has the potential to undermine demonstration of the magnitude of the impact of the recent scaleups of malaria interventions, especially when data are based on presumptive diagnosis of malaria, which has low specificty. Well characterized data from facility-based surveys offer several potential advantages over household surveys and routine facility-based surveillance systems currently being used to evaluate malaria control activities [10]–[11]. A health facility survey can provide information on malaria in different catchment areas of the facility whose collection would have otherwise have required several separate community surveys. Well characterized facility based survey data also provide information on malaria in relation to other diseases and have the potential to detect clustering of malaria cases and pockets of transmission in the locality to guide targeted interventions [7], [8], [9], [12].

Anti-malarial antibody concentrations , show less short-term variation than measurements of parasite prevalence or infection of mosquitoes and may, therefore, be useful for detection of changes in transmission over a longer time. Fitting reverse catalytic model estimates of annual rates of seroconversion of MSP-1_19_ antibodies has been shown to correlate with documented entomological innocualtion rate, and can thus serve as a surrogate measure of transmission intensity [12].

Despite the potential value of health facilitiy surveys, there have been few studies which have assessed the validity of malaria indices collected at health facilities by comparing them with indices obtained during community surveys undertaken in the same areas. To examine the usefulness of health centre surveys as a tool for monitoring area specific malaria epidemiology, we have evaluated malaria specific indices obtained from surveys in health centres in ecologically diverse settings in The Gambia with indices obtained from cross-sectional surveys conducted in their catchment communities.

## Materials and Methods

### Study area

The study was conducted in The Gambia, West Africa. This is a subtropical country at the interface of the Sudan savannah and northern Guinean savannah zones and is characterized by distinct dry and rainy seasons. Average annual rainfall is about 920 mm in the interior and 1450 mm along the coast. The mean annual temperatures ranges from 23° to 27°C along the coast and from 24° to 32° C inland [13]–[14]. Malaria transmission is seasonal, confined largely to the single, short rainy season [15]–[16] and is strongly associated with the prevailing land cover type, transmission being high in the flood plain of the river Gambia [17]–[18].

### Study design

**Figure 1:**
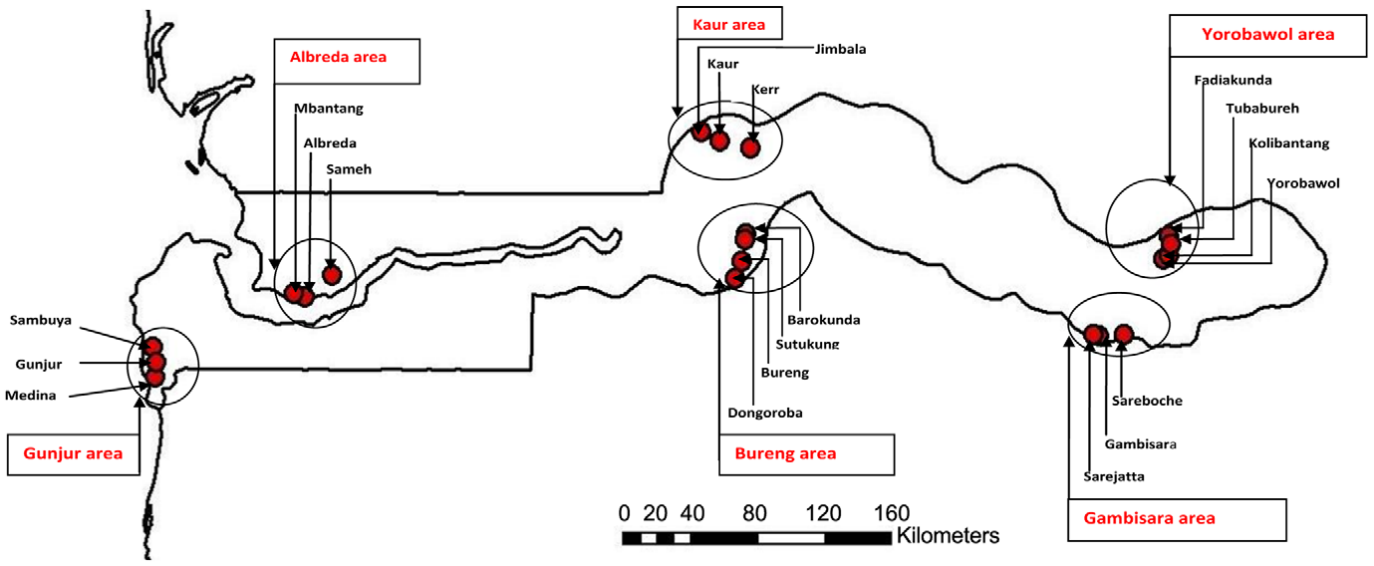
Locations of the catchment villages within the six study areas in The Gambia. Names of health centres and study areas are shown in red and the dots represent the villages in each study area.

The study involved a series of age-stratified, seasonal surveys conducted during the peak rainy season of 2008 and dry season of 2009. Six areas, including two areas from the coast, middle and eastern parts of the country respectively, were studied and at least one area from each of the five administrative divisions of The Gambia was included (Figure 1). One member of each pair of sampled areas was on the north and one on the south bank of the river Gambia. The most centrally located health centre in each area was selected for the facility surveys. The catchment villages of each of the health centres were listed and two villages were selected from this list at random. The selected villages and the one in which the health centre was located were designated as the catchment villages for the community survey. The participants were stratified into five age categories: <2, 2–5, 6–12, 13–25, >25 years. The intention was to recruit 120 persons per age group per survey in each of the six study areas per season.

After approval for the study had been obtained from community leaders and health authorities, sensitisation meetings were conducted to explain the purpose of the study to the study communities. For the community surveys, a list of all the compounds in each selected village was made. These lists were combined into a single list and a sample of 120 compounds selected at random for the community surveys so that the size of each village determined the number of compounds selected from it. The intention was to recruit a person per age group per compound. For the health centres surveys, all patients who attended the study health centres during the study period, irrespective of their age, sex or clinical presentation were eligible to join the study and were invited to participate. No person accompanying a patient to a health centre was enrolled. Patients from whom informed consent was obtained were enrolled consecutively into the study according to their age group until the number required per age group per health centre was achieved.

Inclusion criteria for participation in either kind of survey included having been resident in the study area for a minimum period of four weeks at the time of the survey, willingness to abide by the study protocol, and provision of witnessed individual/parental informed consent.

### Study procedures

Health centre and community survey data were collected at the same period of the year and the same villages, health centres and study procedures were used for both the wet and dry season surveys. A structured questionnaire was administered to each study participant in either kind of survey to collect information on demographic, clinical and socioeconomic characteristics. In addition, bodyweight and axillary temperature were measured.

### Laboratory specimens

Two drops of blood were collected from each participant to prepare two thick films, one drop was collected for determination of haemoglobin concentration, and three drops were put onto filter paper for subsequent malaria antibody measurements. Thick smears were stained with 10% Giemsa and two experienced technicians independently read all the slides. For slides which were positives, malaria parasites were counted against white blood cells (WBC) until 200 WBCs had been counted if more than 10 parasites per field was seen or against 500 WBC if equal or less than 10 parasites per field were counted. The results were quantified as parasites per micro-litre of blood by assuming a total WBC count of 8000 per micro litre of blood. Discrepancies were reviewed by a senior microscopist who was not associated with the study and whose readings were considered final. The same finger prick blood specimens that were used for the malaria slide preparation were also used to estimate haemoglobin concentration employing an automated haemoglobin analyzer - Hemocue® Hb 301 Photometer (Leo Diagnostics, Sweden).

For the serological analyses, reconstituted sera from the filter paper specimens were tested for anti-MSP-1_19_ IgG antibodies by indirect ELISA using the recombinant blood-stage malaria antigen MSP-1_19_, employing previously described protocols [19]–[20]. The duplicate optical densities (ODs) of the ELISA results were averaged and normalised against a positive control. The cut-off for sero-positivity was an OD three standard deviations or more above the mean OD obtained in samples from twenty Europeans who had not been exposed to malaria. Seropositivity was determined only in participants aged twelve months or more. Malaria antibody reactivity was categorized as sero-positive or negative

### Data management and statistical analysis

All data were captured using standard forms designed specifically for this study. All completed forms were checked for internal consistency and queries resolved by the study investigator. Data were double entered and validated in OpenClinica database which is Good Clinical Practice compliant. All statistical analyses were computed using Stata 11 (2009 StataCorp College Station, Texas 77845 USA). All statistical analysis, estimations and hypotheses testing were based on parametric methods. All point estimates of mean and proportions have interval estimates with a 95% confidence interval (95% CI), ranges or interquartile ranges. Equality of means or proportions was tested by t-tests. Proportions or counts were compared by chi-square test, Fisher's exact or two sample tests of proportion. All statistical tests were two sided and statistical significance was set at a p-value of ≤0.05.

Ethical approval for the study and informed consent forms was obtained from the joint MRC/Gambia Government ethics committee. Written informed consent was obtained from all adult study participants and from the parents/guardians of participating children. In addition, assent was obtained from children over 10 years of age.

## Results

A total of 16,230 subjects participated in the study of whom just over a half (53.3%) were recruited from health centres. Approximately half of the participants in the health centre (52.6%) or community (51.0%) surveys were studied in the wet season. Table 1 summarizes the background characteristics of participants in the study. Overall sociodemographic characteristics of study participants were comparable between the study arms. More females than males were recruited during both sets of surveys.

**Table 1:** Background characteristics of the study participants by season and survey.

Variables	Wet season		Dry season	
	Community	Health centre	Community	Health centre
Number enrolled (%)	3870 (23.8)	4543 (28.0)	3716 (22.9)	4101 (25.3)
Average age in years (SD)	16.4 (18.8)	15.5 (17.2)	16.2 (18.3)	15.7 (17.6)
Number of females (%)	2063 (53.5)	2637 (58.1)	1956 (52.7)	2369 (58.4)
Number <5 years (%)	1337 (34.6)	1675 (36.9)	1284 (34.6)	1489 (36.5)
**Ethnic groups**,n (%)				
Mandinka	2243 (58.1)	1723 (37.9)	2057 (55.4)	1615 (39.9)
Fula	960 (25.8)	1454 (32.0)	1023 (27.6)	1256 (30.8)
Wollof	295 (7.6)	516 (11.4)	276 (7.4)	413 (10.1)
Others	326 (8.5)	849 (18.7)	355 (9.6)	793 (19.5)
*P. falciparum*, n (%)	478 (12.4)	1088 (24.0)	80 (2.2)	46 (1.1)
Sexual stage parasites, n (%)	56 (1.5)	65 (1.4)	7 (0.2)	4 (0.1)
Anti MSP1_19_ positivity, n (%)	736 (20.9)	1122 (33.2)	712 (21.0)	696 (20.7)
Mean temperature °C(SD)	36.8 (0.5)	37.3 (1.0)	36.7 (0.4)	36.9 (8.0)
Fever (≥37.5°C), n (%)	214 (5.5)	1410 (31.4)	83 (2.2)	782 (19.2)
Mean haemoglobin (g/dL)(SD)	11.1 (2.0)	11.0 (2.7)	11.6 (1.8)	11.0 (2.1)
Anaemia (Hb≤8 g/dL), n (%)	283 (7.4)	440 (10.0)	127 (3.4)	317 (8.0)
Slept under bednet ,n(%)	3348 (86.8)	3568 (78.6)	2934 (79.3)	2848 (69.8)

### *Plasmodium falciparum* asexual stage parasitaemia

**Figure 2:**
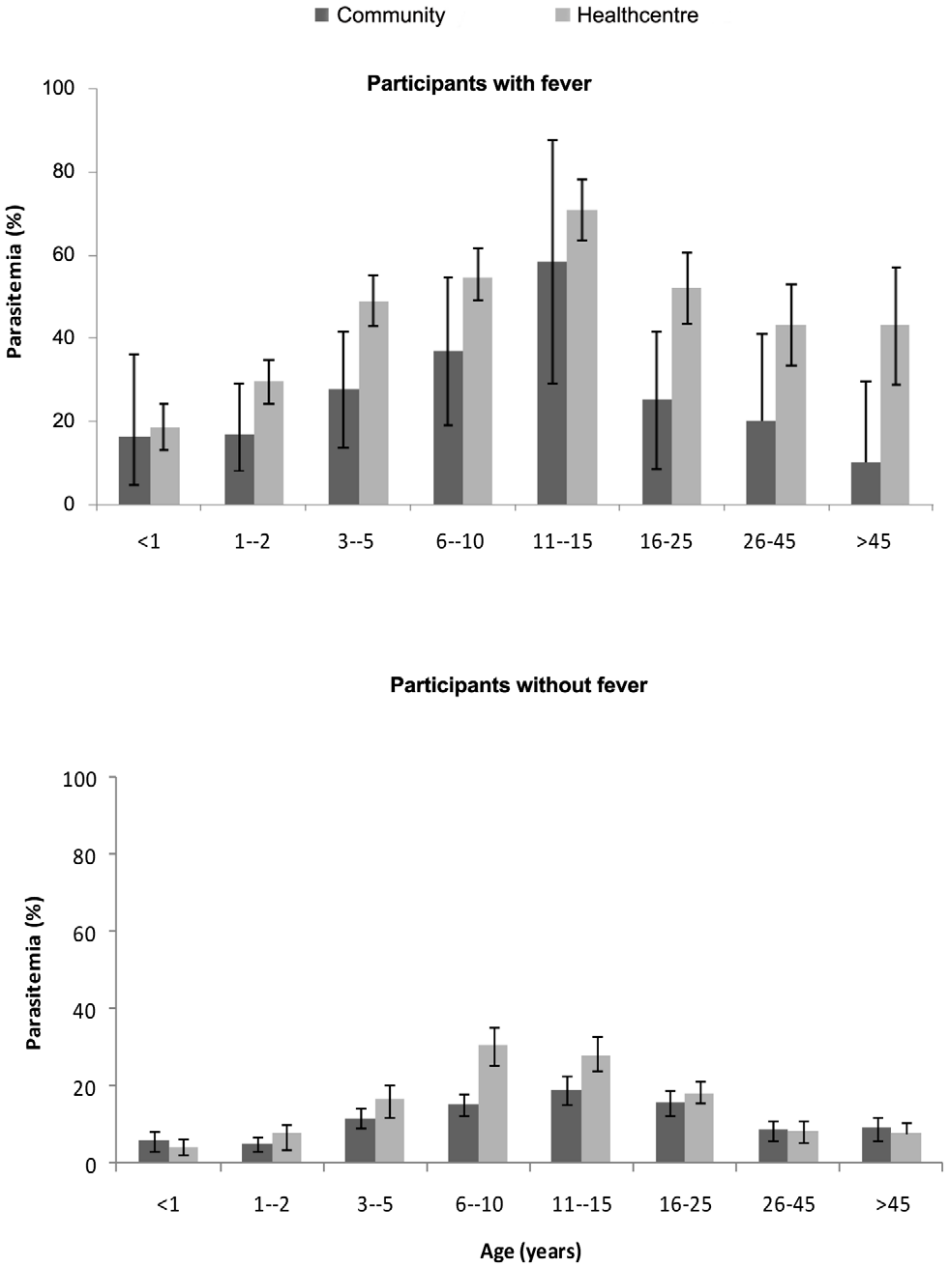
The age pattern of asexual *P. falciparum* malaria parasitaemia in febrile and nonfebrile participants. The horizontal axis shows the age groups and the vertical axis shows prevalence of parasitaemia in each age group. Percentage and 95% confidence intervals of parasite prevalence are shown by the bars.

**Figure 3:**
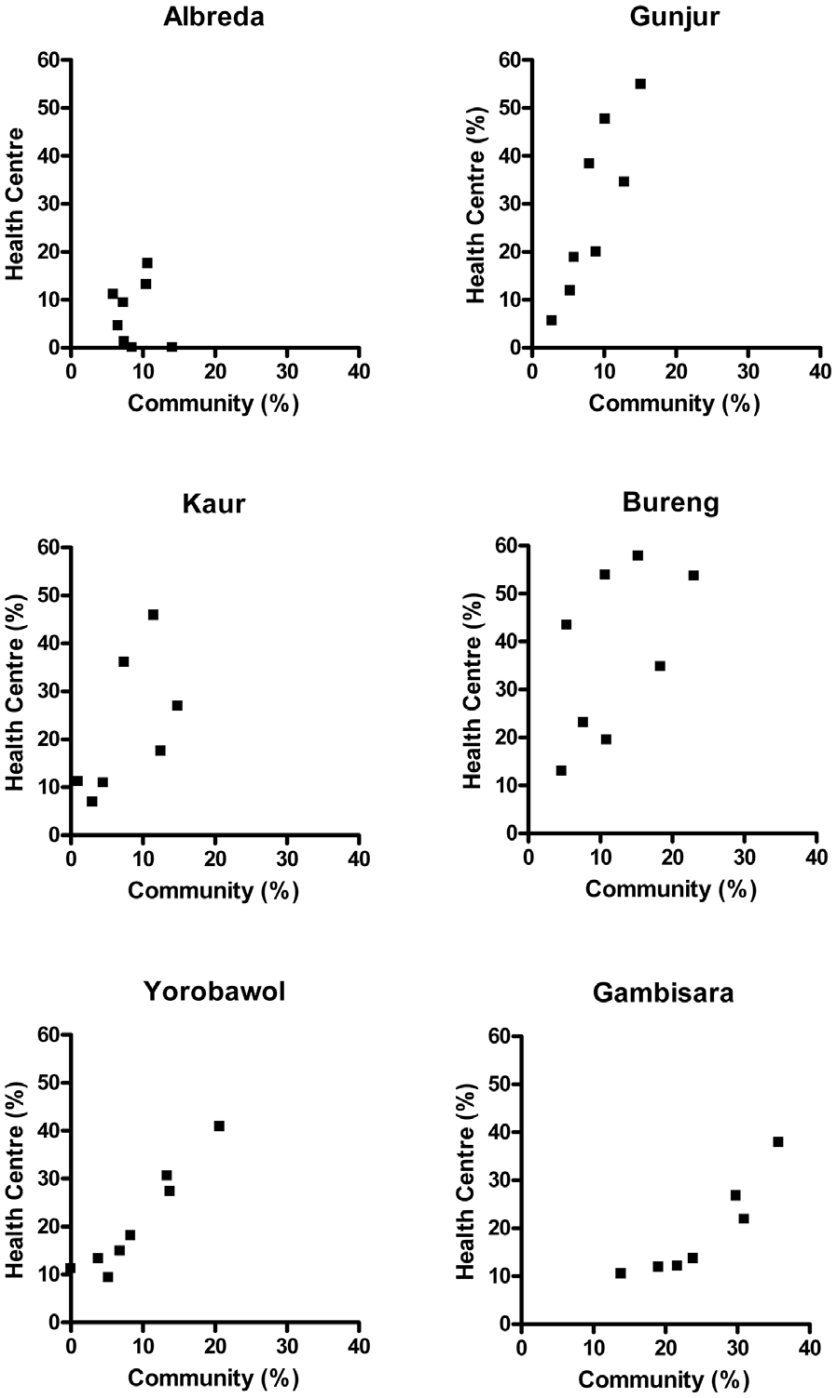
Correlation between age specific parasite prevalence in the community and health centre surveys. This was in the six study areas during the wet malaria transmission season in The Gambia (agegroups: <12, 12-, 36-, 72-,132-,192-, 312-, 552- months).

**Figure 4:**
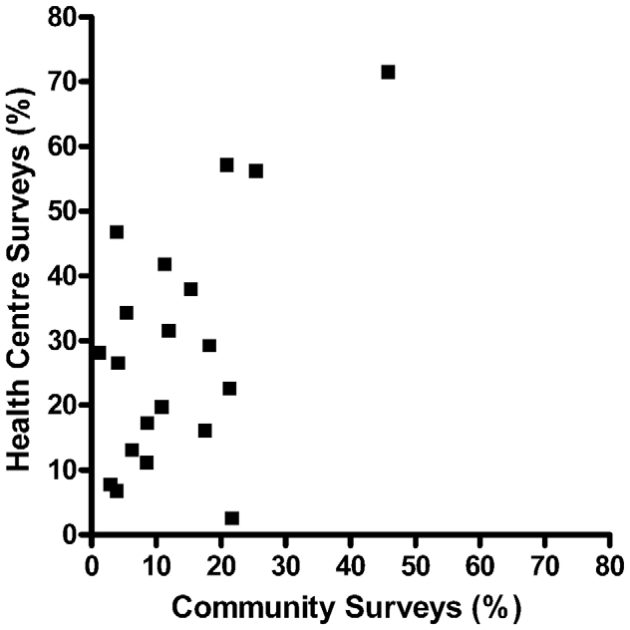
Relationship between village specifc parasite prevalence in the community and health centre surveys durng the wet malaria transmission season. Data points are parasite prevalence in each of the twenty villages used in the study. Some of the ‘village’ data that have been obtained from the heatlth facilities were quite small so that the correlation here has more scatter than it would do if the health-centre sampling had got optimal number per village.

During the rainy season, *P. falciparum* asexual stage parasitaemia was much higher in subjects seen in the health centre (24.0% [1088/4543]) than in the community surveys (12.4% [478/3870) (OR = 2.2; 95% CI 1.9, 2.5). In the dry season the prevalence in both groups was very low and, surprisingly, even lower in the health centre than in the community surveys (1.1% vs. 2.2% respectively; OR = 0.51; 95% CI 0.34, 0.75). Table 2 presents the prevalence of malaria by age, season and type of survey. In both surveys, parasite prevalence increased with age, reaching a peak in 11–15 year olds and thereafter declined with increasing age (Figure 2). Adolescent males had a higher prevalence of parasitaemia than adolescent females but this difference reached statistical significance only in the health centre surveys. Adolescent males participating in the community or health centre surveys were less likely than females to sleep under a mosquito net (OR = 0.28; 0.22, 0.35 and OR = 0.48; 0.39, 0.60 respectively). Correlation between age specific parasite prevalence rates in the community and health centre surveys in the six study areas in the wet season are presented in Figure 3. In addition, figure 4 presents the relationship between village specific parasite prevalence rates by community surveys and health centre surveys in the twenty villages in the six study areas. The overall correlation between health centre and community survey data for age specific parasite prevalence rates was stronger in the wet season (R^2^ = 0.83) than in the dry season (R^2^ = 0.42). Parasite density varied with age in both sets of surveys. In the health centre surveys, it peaked in early childhood (3–5 year olds) with a mean density of 23000/uL. In contrast, in the community surveys it peaked in adolescents (11–15 year olds) with a peak density of only 370/uL.

**Table 2:** Prevalence of malaria parasitaemia in The Gambia by age, season and survey.

Variables	Wet season, N (%)		Dry season, N (%)	
Age (years)	Community	Health centre	Community	Health centre
<1	301 (5.3)	517 (9.3)	322 (0.6)	453 (1.1)
1–2	558 (7.0)	725 (16.4)	545 (1.3)	663 (1.2)
3–5	653 (12.6)	583 (31.2)	550 (1.8)	543 (1.1)
6–10	598 (16.4)	636 (40.3)	581 (4.7)	575 (2.6)
11–15	452 (19.9)	441 (42.2)	511 (4.1)	375 (0.8)
16–25	478 (16.3)	650 (24.9)	416 (2.6)	590 (1.2)
25–45	410 (9.0)	633 (14.2)	393 (0.5)	544 (0.2)
>45	410 (9.0)	358 (12.6)	389 (0.0)	334 (0.0)

### *P. falciparum* gametocytaemia

The prevalence of *P. falciparum* gametocytaemia was similar in subjects seen in the community or in the health centre surveys in the wet season (1.5% [56/3870] vs. 1.4% [65/4543] respectively; P = 0.97) and in the dry season (0.2% [7/3716] vs. 0.1[4/4101] respectively; P = 0.44). The highest gametocyte carriage rates were seen in 6–12 year olds participating in the community or in the health facility surveys (2.2% and 2.5% respectively). The age pattern of gametocyte prevalence was similar to that seen for asexual parasites. None of the differences in gametocytes prevalence between age groups reached statistical significance in either of the two sets of surveys .

### Anti-MSP-1_19_ seroprevalence

**Figure 5:**
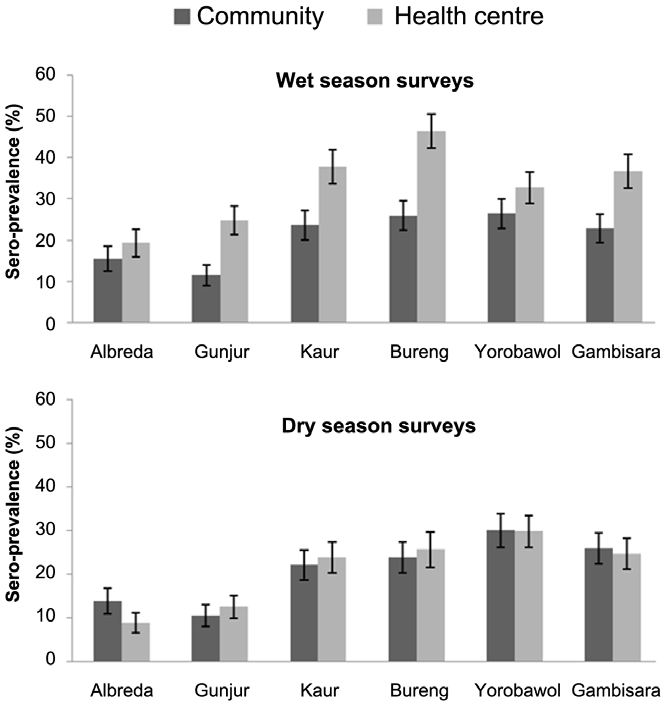
Seroprevalence by season in subjects seen during the health centre or community surveys. The horizontal axis shows the study areas and the vertical axis show the proportion of sero-positive individuals (%) in each study area. Percentage and 95% confidence intervals of seroprevalence are shown by the bars.

**Figure 6:**
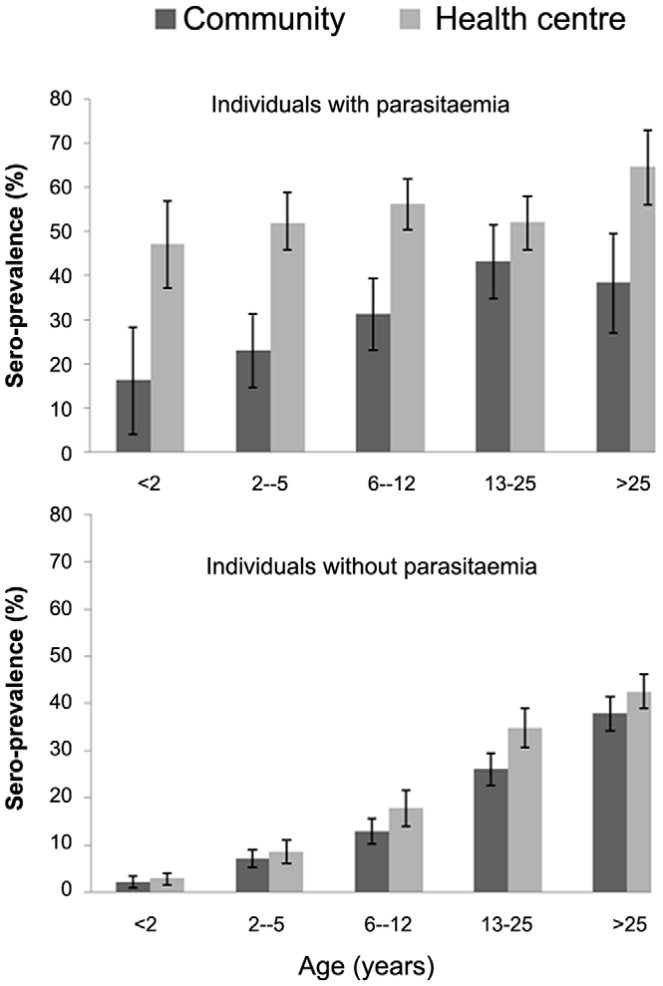
Age specific seroprevalence in participants with and without patent parasitemia seen during the health centre or community surveys. The horizontal axis shows the age groups and the vertical axis shows seropositive individuals (%) in each age group. Percentage and 95% confidence intervals of seroprevalence are shown by the bars.

Table 3 summarizes the proportions of subjects sero-positive for antibodies to MSP-1_19_ by their background characteristics and by season and type of survey. Overall, seroprevalence was higher in subjects seen in the health centre surveys (33.2%) compared with those seen in the community surveys (20.9%) in the wet season but prevalences were similar in the two groups during the dry season (20.7% versus 21.0%; P = 0.73) (Figure 5). There was a considerable difference in the prevalence of seropositivity between villages (Figure 5). Seropositivity increased with age in all surveys and seroprevalence was higher in participants with patent parasitemia than in subjects without parasitaemia during either the community surveys or health centre surveys (33.4% vs. 19.9%; P<0.001 and 54.1% vs. 22.3%; P<0.001 respectively) (Figure 6). Multivariate logistic regression analyses adjusting for age, study area and type of survey showed that sex (OR = 1.2; 1.08,1.30; P<0.0001), ethnicity (OR = 1.1; 1.02,1.08; P = 0.002) and patent malaria infection (OR = 3.8; 3.39,4.41; P<0.0001) were significantly associated with seropositivity.

**Table 3:** Proportion of subjects seropositive for antibodies to MSP-1_19_ by background characteristics of participants, season and type of survey.

Variables	Wet season sero-positives,% (95%CI)		Dry season sero-positives,% (95%CI)	
	Community	Health centre	Community	Health centre
**Gender**				
Males	18.9 (16.9,20.8)	29.0 (26.6,31.4)	18.9 (16.9,20.8)	14.3 (12.4,16.2)
Females	22.7 (20.8,24.6)	36.1 (33.9,38.2)	22.9 (21.0,24.9)	25.3 (23.4,27.2)
**Fever**				
Yes	21.5 (15.8,28.1)	36.6 (33.6,39.6)	11.3 (4.9,21.0)	14.6 (11.9,17.6)
No	20.9 (19.5,22.3)	31.6 (29.7,33.5)	21.2 (19.8,22.6)	22.1 (20.5,23.6)
**Patent Parasitaemia**				
Yes	33.4 (29.0,37.7)	54.8 (51.6,57.9)	33.3 (22.8,43.8)	33.3 (18.9,51.6)
No	19.1 (17.6, 20.4)	24.7 (23.0,26.4)	20.7 (19.3,22.1)	20.6 (19.2,21.9)
**Gametocytaemia**				
Yes	40.7 (27.5,53.9)	70.0 (57.1,82.8)	28.6 (0.0, 64.7)	33.3 (0.0,98.6)
No	20.6 (19.2,21.9)	32.6 (31.0,34.1)	21.0 (19.6,22.4)	20.7 (19.3,22.0)
**Slept under a bednet**				
Yes	20.6 (19.1,22.0)	31.0 (29.2,32.70	19.8 (18.2,21.3)	19.0 (17.4,20.6)
No	22.8 (19.0,26.5)	40.7 (37.1,44.2)	25.4 (22.2,28.5)	24.4 (21.8,27.0)
**Ethnic groups**				
Fula	27.6 (24.7,30.6)	44.8 (41.8,47.8)	29.7 (26.8,32.7)	33.5 (30.7,36.5)
Wolof	18.8 (14.1,23.9)	38.2 (33.2,43.2)	19.5 (14.8,24.9)	16.2 (12.3,20.6)
Mandinka	19.0 (17.3,20.8)	26.3 (23.9,28.7)	17.0 (15.3,18.7)	14.0 (12.2,16.0)
Others	15.2 (11.1,19.8)	24.7 (21.3,28.2)	19.9 (15.5,24.7)	17.1 (14.4,20.1)

### Haemoglobin and anaemia

The mean haemoglobin concentration obtained in study subjects seen in the community or health centre surveys was similar in the wet season (11.05 g/dl versus 11.02 g/dl respectively P>0.05) but was significantly higher in subjects seen in the community than in the health centre surveys in the dry season (11.61 g/dl versus 11.01 g/dl respectively P<0.001). In both surveys, a low mean haemoglobin concentration was associated with being female, parasitaemic, less than 6 years of age and living in the eastern part of The Gambia. The population attributable proportion of anaemia (Hb≤8.0 g/dl) due to malaria was low but was about three times higher in the health centre (15.6%) than in the community (5.4%) surveys in the wet season. In the dry season it was similar (<1%) in both surveys. Anaemia associated with malaria was most prevalent in children under five years and decreased with increasing age in subjects seen in either the health centre or in the community surveys.

### Fever (temperature ≥37.5° C)

The prevalence of fever (temperature ≥37.5° C) was higher in subjects seen during the health centre surveys, (31.4% [1410/4497]) compared with those seen in the community surveys (5.5% [214/3860]) in the wet season (P<0.001). The prevalence of fever was 19.2% (782/4069) in subjects seen in the health centre surveys and 2.2% (83/3708) in those seen in the community surveys in the dry season (P<0.001). In both surveys the prevalence of fever was highest in children under five years of age. The proportion of fevers associated with malaria parasitaemia was however significantly higher in subjects seen in the health centre surveys (44% [619/1410]) than in those seen during the community surveys (25% [53/214]) in the wet season (P<0.0001). In the dry season, the percentage of subjects with fever associated with parasitemia seen during the health centre surveys (1.5% [12/782])or community surveys (3.6% [3/83]) were not significantly different (P = 0.16).

The proportion of fevers attributable to malaria was 12.7% in subjects seen during the community surveys compared with 26.4% among those seen in the health cnetres surveys in the wet season. In the dry season, the proportion was 1.3% in those seen in the community surveys compared with 0.5% in those seen in the health centre surveys. The proportion of fevers attributable to malaria peaked earlier in subjects seen in the community surveys (6–12 year olds) than in those seen during the health centre surveys (13–25 year olds).

## Discussion

Health facility data are easier to collect than community survey data and could allow effective monitoring of malaria control activities provided that they are representative and accurate [8]–[9]. One facility survey can provide information on the pattern of malaria in different catchment communities of the health facility, collection of which would otherwise require separate surveys in each of these communities. In this study, data from community and health centre surveys have been used to assess the current level of malaria endemicity in The Gambia. We naturally anticipated a higher prevalence of fever, anaemia and parasitaemia in the health centre data. However data from both settings indicate a significant decline in malaria transmission from previous years, even though the disease remains endemic throughout The Gambia. There was a strong relationship between malaria indices obtained in the community and health centre surveys undertaken in most areas from different ecologies, with some important exceptions which are not easily explained and may require further studies.

Age patterns of malaria infection, disease and mortality are determined by the intensity of malaria transmission [21]. The age pattern of malaria infection and of cases of clinical malaria is, therefore, an important index for measuring area specific transmission intensity. Successful malaria programmes are currently leading to changes in the age distribution of malaria and well characterized age patterns may help to evaluate future changes [2].

In our study, the age pattern of parasitaemia did not differ significantly between subjects seen during community or health centre surveys and varied little by area, season, sex or clinical status. In the dry season parasite prevalence was higher in the community than in the health centre surveys. Though the absolute difference was of a small magnitude (1.1%), the relative difference was significant and of a similar magnitude but opposite effect size to that noted in the wet season. The implication may be that in low transmission settings or where transmission is highly seasonal using dry season health centre data may overestimate the effects of malaria control measures except that this becomes less significant when there is very low prevalence levels as occur in dry season.

Both surveys showed a peak of malaria infection in young adolescents, consistent with a lower endemicity than existed in previous decades [22]. This age shift has public health implications, as malaria control activities previously focussed on young children but more attention now needs to be given to older children and adolescents. Thus, the current recommendation for universal coverage with ITNs and other antimalarials is an appropriate priority for this population [1], [23].

Monitoring malaria transmission over time is necessary to assess the long-term impact of control activities on transmission intensity, disease and mortality [21]. The standard for measuring transmission intensity, the entomological inoculation rate, lacks precision and is too expensive to be a widely used public health tool. Serological methods have been shown to be a robust and sensitive alternative way of measuring changes in transmission [19]–[20]. In this study, the usefullnes of seroprevalence data obtained from health centres surveys was examined. Our results showed that seroprevalence was higher in subjects seen during the health centre surveys than in those seen during community surveys in the wet season but not in the dry season. This suggests that acute malaria occurring in the wet season is the main reason for the higher seroprevalence in the health centre surveys consistent with an earlier study that showed that acute rather than chronic infection is the dominant factor in antibody acquisition [24]. Dry season health centre serological measurements may be more useful for comparing transmission intensity across different settings than those obtained during the malaria transmission season.

In many malaria endemic countries, fever has been used as a surrogate for malaria, particularly in children under five years of age [25]. The burden of malaria has often been estimated using presumptive diagnoses based on fever [1]. Intense malaria transmission leads to high immunity and an increased proportion of asymptomatic compared with symptomatic malaria infections [26]–[27]. As a consequence, a change in the fraction of fevers attributable to malaria implies change in transmission. In this study, the attributable fraction was lowest in children under two years both in the community and health centre surveys. The proportions of fever due to malaria in children <5 years were 11% in the community and 17% in the health centre surveys in the wet season. The situation was even more striking in the health centres in the dry season surveys where the prevalence of fever was 19% and only 2% of these fevers was associated with malaria. Treating such patients with anti-malarial drugs, for example with an ACT, is a waste of scarce resource [23].

In conclusion, carefully conducted health centre surveys have great potential as a surveillance tool for evaluating area specific malaria control activities and for monitoring changes in local malaria epidemiology. The potential for using such an approach will be improved by longitudinal studies that enable the continuous validation of health centre data. Additional information on the characteristics of health facilities and health seeking practices of the catchment population may further help to interpret the information obtained.
